# Community stigma endorsement and voluntary counseling and testing behavior and attitudes among female heads of household in Zambézia Province, Mozambique

**DOI:** 10.1186/1471-2458-13-1155

**Published:** 2013-12-10

**Authors:** Abraham Mukolo, Meridith Blevins, Bart Victor, Heather N Paulin, Lara ME Vaz, Mohsin Sidat, Alfredo E Vergara

**Affiliations:** 1 Vanderbilt Institute for Global Health, Vanderbilt University School of Medicine, Nashville, USA; 2 Friends in Global Health, Maputo, Mozambique; 3 Department of Community Health, University Eduardo Mondlane, Maputo, Mozambique; 4 Owen Graduate School of Management, Vanderbilt University, Nashville, USA; 5 Division of Infectious Diseases, Vanderbilt University School of Medicine, Nashville, USA

**Keywords:** HIV-related stigma, Voluntary counseling and testing, Women, Negative labeling and devaluation, Social exclusion, Mozambique

## Abstract

**Background:**

Some aspects of HIV-related stigma have been shown to be a barrier to HIV services uptake and adherence to antiretroviral treatment (ART). Distinguishing which domains of stigma impact HIV services uptake can enhance the efficacy and efficiency of stigma-reduction interventions.

**Methods:**

The relationships between use of voluntary counseling and testing (VCT) services and two domains of community stigma identified through factor analysis, negative labeling/devaluation and social exclusion, were investigated among 3749 female heads of household. Data were from a general household survey conducted in rural Mozambique. Multivariable logistic regression outcomes were: lifetime VCT use, past-6-months VCT use and VCT endorsement.

**Results:**

Thirteen percent (13%) of the participants reported lifetime VCT use, 10% reported past-6-months VCT use and 63% endorsed VCT. A 25-point decrease (from 50 to 25) in the score for negative labeling and devaluation stigma was associated with increased lifetime VCT use (adjusted OR: 1.6, 95% CI: 1.1-2.3) and past-6-months VCT use (adjusted OR: 1.6, 95% CI: 1.1-2.4). A decrease from 50 to 25-points in the score for social exclusion stigma was associated with 1.5 and 1.3-fold increase in odds for past-6-months VCT use and endorsing VCT use, respectively (*p* < 0.001 for both). Compared with never-testers, considerably high endorsement of VCT use was observed among testers who did not receive HIV test results (adjusted OR: 2.7, 95% CI: 1.6-4.6) and much higher among testers who received results (adjusted OR: 7.3, 95% CI: 4.9-11.0). Distance from health facilities was associated with lower VCT use, but not lower endorsement of VCT.

**Conclusions:**

VCT use and endorsement might differ by domains of stigma held by individuals in the community. Greater uptake and favorable disposition towards use of VCT services in rural settings might be achieved by addressing stigma via domain-specific interventions and by improving the proximity of services and the dissemination of HIV test results.

## Background

Community attitudes towards people living with HIV/AIDS impact the utilization of HIV care and treatment services [1,2]. An increase in knowledge about HIV transmission, prevention and treatment that is created through the increased availability of HIV care and treatment services, has been shown to diminish negative community attitudes [3]. This is particularly relevant if misinformation and ‘erroneous’ beliefs about HIV/AIDS are reduced in the process of service uptake [4]. However, HIV stigma seems to persist as a barrier to HIV services uptake and patients’ adherence to antiretroviral treatment (ART) despite the scale-up of HIV care and treatment services [1,5-7].

Research has shown stigma to be a multidimensional construct [8-10] and to be manifested in a variety of contexts [11]. The literature on HIV-related stigma has identified some key domains of stigma, such as negative labeling, devaluation, status loss and social exclusion, that are observable at the community (or public), institutional and intrapersonal levels [8,12,13]. More precise specification of the effects that these individual domains of stigma have on the use and endorsement of voluntary counseling and testing (VCT) services is needed, in order to develop effective (targeted) stigma reduction strategies [5,6]. Furthermore, while the advantages of investigating stigma in varying contexts of HIV knowledge and public health response to the HIV epidemic have been suggested [14], domain-specific literature is sparse. Negative social norms that are created and maintained at a community level, such as community stigma, are likely to influence the use of HIV services by individuals [3]. For example, poor adherence to HIV treatment tends to be high among patients who think HIV is highly stigmatized in their community [1,15]. Thus, one’s endorsement of community stigma would influence her behavior and attitudes regarding the use of VCT.

Using data from female heads of household in a rural setting in Mozambique, we examined the relationship between endorsement of two domains of community stigma identified through factor analysis — negative labeling/devaluation (NLD) and social exclusion (SoE) — and these women’s participation in and attitudes toward VCT services. As in many high HIV/AIDS prevalence areas, women between the ages of 15 and 45 years are targeted under interventions to scale up the prevention of mother-to-child transmission (PMTCT) of HIV in Mozambique [16-19]. The questions we addressed are: Does endorsement of community stigma have a significant association with VCT use and endorsement? If an association is present, is it significant after adjusting for increased knowledge about HIV/AIDS and service provision barriers? Do these associations significantly differ by domains of community stigma?

## Methods

### Survey background and design

A population-based survey called the *Ogumaniha*-SCIP survey was conducted in late 2010 among 3749 female heads of household in 259 randomly selected enumeration areas across 14 districts of Zambézia Province, Mozambique [20]. In the Echuabo language, *Ogumaniha* means “united/integrated for a common purpose”; SCIP stands for “Strengthening Communities through Integrated Programming,” which is a project funded by the United States Agency for International Development (USAID) and implemented by a consortium of partners led by World Vision, Inc. Data were collected in two phases: first, we collected a Zambézia-wide sample to provide province-wide estimates, then we focused on three districts [20] to generate more precise baseline estimates. Interviews covered various topics, including socio-demographics, access to healthcare, HIV knowledge and attitudes towards people living with HIV/AIDS (PLWHA). Details about sampling, data collection and management protocols have been published elsewhere [20]. Approximately 99.1% of all households approached consented to participate in the study, which was approved by the Mozambican national bioethics committee and the Institutional Review Board at Vanderbilt University.

### VCT outcomes

We examined three aspects of voluntary counseling and testing (VCT) use, including overall or “lifetime” VCT use, VCT use during the 6 months before the interview, and general endorsement of VCT use. Overall VCT use was assessed using data from responses to the following questions: (1) ‘Have you received voluntary counseling and testing (VCT) in the past 6 months?’ and (2) ‘Have you ever received voluntary counseling and testing (VCT) at any time during your life prior to the last 6 months?’ A “lifetime VCT use” score was generated by combining responses to both questions, such that responding affirmatively to either question indicated lifetime VCT use. A “past-6-months VCT use” score was also analyzed separately. Attitudes about VCT use were assessed by generating a “VCT endorsement” score based on responses to the question: ‘Do you think it is worthwhile to receive voluntary counseling and testing and learn your HIV status?’ The skip pattern in the survey was structured such that this question was posed only to participants who responded affirmatively to the question: ‘Have you ever heard of VCT services?’ All three aspects of VCT use, “lifetime VCT use”, “past-6-months VCT use” and “VCT endorsement” were analyzed as binary variables (1 = used or worthwhile; 0 = not used or not worthwhile).

### Stigma measurement

Stigma items were adapted from a questionnaire used by Pulerwitz et al. [2,21], which lists 15 items reflecting stigmatizing attitudes, beliefs and behaviors that a participant endorses on a 4 point Likert scale from “strongly disagree” to “strongly agree.” The statements reflect labels and stereotypes that devalue and reduce a person with HIV to a tainted and socially undesirable status [8,10,22,23], as well as specific exclusionary actions towards PLWHA (see Additional file 1 for details). The original stigma measure [2] was modified to tailor questions to our sample of female heads of household. Two dimensions of stigma, negative labeling/devaluation (NLD) and social exclusion (SoE), were generated through factor analysis of the 15 stigma items, specifically principal components analysis, with orthogonal varimax rotation, by using STATA 11.2 (http://www.stata.com). Cronbach alphas were 0.74 for NLD and 0.73 for SoE, which explained 94.7% of the variance. These Cronbach alphas were comparable to those reported by Pulerwitz et al. (α = 0.76 for the combined 15-item scale) [2,21], indicating acceptable reliability in our context. Items in each dimension of stigma were scored such that higher scores denoted greater stigma. Scales for each dimension were calculated by taking the mean value of non-missing items and then normalized to a 0–100 range.

### Other correlates

Several other expected correlates of HIV services uptake were considered, including participant’s age, years of education, marital status, distance from the clinic/health facility, living in an isolated district versus living in a district close to the provincial capital, religious affiliation, knowledge of HIV transmission, perceived risk of HIV infection, experience/familiarity with HIV infection, belief in ART efficacy (as proxy for being favorably disposed to HIV care and treatment services), healthcare services access/contact and health-related quality of life (as proxy for self-evaluated state of health). State of health was considered because the literature suggests that perceived ill-health likely motivates HIV testing [24]. Past testing behavior has been found to predict current and future testing behavior in a similar population [25]. Thus, for attitudes towards VCT use, the impact of receiving versus not receiving HIV test results at the last VCT visit was also considered, as it assesses the quality of past experience with VCT. We assumed that not receiving test results after completing VCT would be negatively associated with attitudes towards VCT, whereas receiving results would have no association, because it is an expected outcome of VCT.

*Knowledge about HIV transmission* was measured by the number of correct responses to questions regarding HIV transmission routes and ways to prevent them. We assessed five domains of knowledge, including knowledge typically covered in public health education campaigns and targeted health education programs conducted in health centers and community settings. The focus was on knowledge about adult-to-adult and mother-to-child transmission routes and transmission via casual contact (see Additional file 1 for details). A count of correct responses was taken for each of the five questions and categorized as follows: 0 = No correct responses, 1 = One correct response and 2 = Two or more correct responses. A summative score (range: 0–10 points) was generated with higher scores indicating higher (and better) knowledge.

*Perceived risk of HIV infection*, which is known to influence HIV testing behavior, was assessed by asking: ‘What are the chances you might become infected with HIV?’ Responses options were coded as follows: 1 = No chance, 2 = Small chance, 3 = Good chance and 4 = Already infected. Non-response or responses of “Don’t know” were also recorded. Perceived risk was treated as a categorical variable with non-responses and “Don’t know” responses collapsed into a single category.

*Experience/familiarity with HIV infection* was assessed through participants’ direct experience with HIV infection, which has been shown to moderate attitudes towards PLWHA, consistent with findings about other socially stigmatized conditions like mental illness [11]. Participants were considered to have direct experience/familiarity with HIV infection if they gave a positive response to at least one of the following questions: ‘Do you have relatives with HIV?’, ‘Do you have friends with HIV?’ or ‘Do you have HIV?’ For ethical and other considerations, since the last question was posed to women who reported using PMTCT services, responses were validated by comparing the total number of positive responses with the number reporting that they were already infected when asked: ‘What are the chances that you might become infected with HIV?’.

*Belief about the efficacy of ART* was assessed by asking: ‘Do you think antiretroviral treatment helps people with HIV to be healthier?’ and ‘Do you think alternative treatments available in the community or from traditional healers can help people with HIV?’ Each item was treated as a distinct binary variable in the analyses.

*Healthcare services access/contact* was assessed by asking about three different healthcare systems that participants could utilize: government health centers or hospitals, private pharmacies and traditional healers. Each item was treated as a distinct binary variable in the analyses. The frequency of visits to each system was not estimated due to data reliability issues.

*Quality of life* (QoL) was assessed using an adapted version of the WHOQOL-BREF, a 31 item measure of subjective QoL in 6 domains, including physical well-being, psychological well-being, independence, social relationships, environment and spirituality. The scale has been shown to have good psychometric properties in a similar population from Botswana (physical well-being α = 0.74, psychological well-being α = 0.53, independence α = 0.72, social relationships α = 0.71, environment α = 0.73, spirituality α = 0.47) [26]. Ten of the 31 items deemed relevant to the cultural context of Mozambique were selected (see Additional file 1 for the list). Factor analysis revealed that these 10 items capture two distinct dimensions of QoL that are consistent with the literature [27]: QoL-chronic (or long-term well-being) and QoL-acute (short-term well-being). QoL-chronic (α = 0.83) consists of the two global health ratings, plus daily living, capacity to work, access to health treatment and mobility. QoL-acute (α = 0.68) includes physical pain, dependence on medication/treatment, energy/vitality and negative feelings. Only the QoL-chronic scale was used in subsequent analyses, since lifetime VCT use was being examined.

### Statistical methods

**Figure 1:**
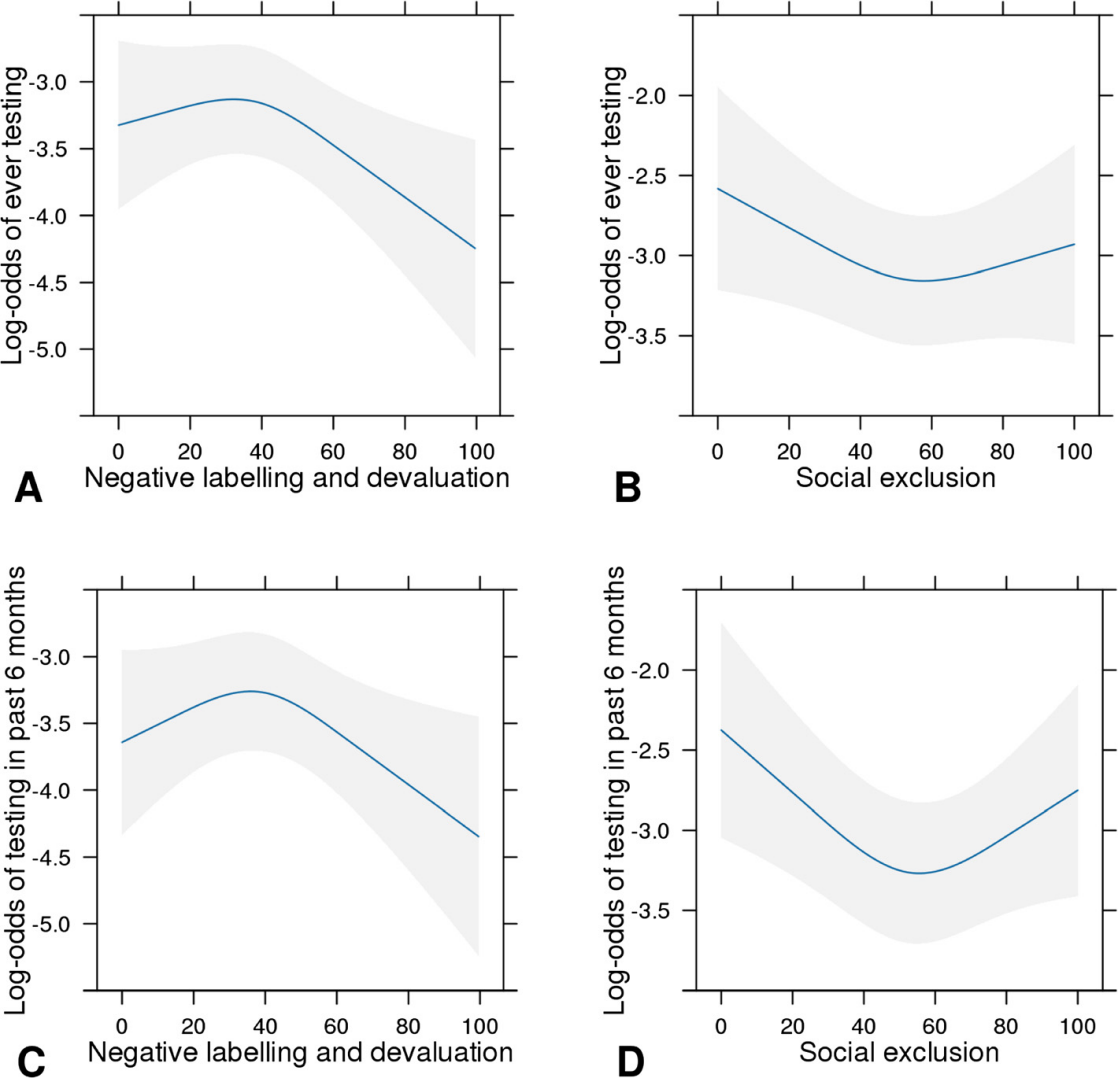
**Marginal log-odds of HIV testing for negative labeling and devaluation stigma and social exclusion.** Covariate-adjusted log-odds (adjustment values are medians or categories with largest proportions) of ever testing **(Panels A and B)** or testing during the past six months **(Panels C and D)** for negative labeling and devaluation stigma **(Panels A and C)** and social exclusion stigma **(Panels B and D)**.

We modeled the probability of our VCT outcomes, lifetime VCT use, past-6-months VCT use and VCT endorsement, by using multivariable logistic regression. The primary predictors were the two dimensions of community stigma, NLD and SoE. We used a regression spline model [28,29] which predicts each VCT outcome from several independent variables because some predictor variables, like NLD and SoE, had curvilinear distributions. For example, the curvilinear relationship between stigma scales and the VCT use scores can be seen in Figure 1. When there was evidence of nonlinearity, continuous variables were modeled using restricted cubic splines [28]. Thus, the two stigma variables (NLD and SoE) enter each model as nonlinear, continuous variables. The restricted cubic splines method ensures that the distributions of the stigma variables entered in the model fit the actual stigma data. Rather than forcing stigma variables to be linear, their functions/distributions are more appropriately represented by knots whose positions are “determined automatically according to equally spaced centiles of the distribution” [29]. In our analyses four knots were placed at the 5th, 35th, 65th and 95th percentiles of the distribution [28]. Multiple imputation was used to account for missing values for baseline predictors and to prevent case-wise deletion of missing data. We used the functions ‘aregImpute’ and ‘fit.mult.impute’ in the Hmisc package of R, which uses predictive mean matching to take random draws from imputation models; 10 imputation data sets were used in the analysis. Covariates were selected *a priori,* including knowledge about HIV/AIDS transmission and treatment, perceived risk of HIV infection, healthcare service access/contact and socio-demographic factors typically associated with VCT use behaviors and attitudes, such as age, education and marital status [30]. Wald statistics were used to test the significance of linearity, interaction, and covariate effects in each of the three outcome models [31]. When significant, we also included an interaction effect between both stigma scales. All hypothesis testing was two-sided with a level of significance set at 0.05. We employed R-software 2.13.1 (http://www.r-project.org) for all data analyses.

## Results

Table 1 shows the characteristics of the study population. Of the 3749 female heads of household interviewed, 489 (13%) reported lifetime voluntary counseling and testing (VCT) use and 374 (10%) reported past-6-months VCT use. Data about the importance of VCT was available for 1069 participants, i.e., those who responded affirmatively when asked if they knew about the existence of VCT services and had no missing data on the importance of VCT. Of these, 676 (63.2%) agreed with the statement that it is worthwhile to use VCT and learn about your HIV status. The median age of participants (n = 3749) was 29 years (interquartile range (IQR): 23–36 years) and did not differ by level of stigma (for either stigma scale). Half of the participants had at least 2 years of education and less than 25% had more than 4 years of education; 57% resided in districts regarded as isolated. Approximately 75% said they were married or in common-law relationships, and 17% said they were single. Religious affiliations showed significant diversity: 47% were Catholic, 34% were non-Catholic Christians, 9% were Muslim and about 10% were other religions. Less that 50% were fluent in Portuguese, the country’s official language. The average distance from the center of the enumeration areas to the nearest public health facility was 6.2 km (IQR: 3.2-10.3 km). About 48% were unaware of their HIV infection risk, 25% were confident that they were not at any risk of HIV infection, while 25% thought they were at risk of being infected with HIV. About 2% disclosed that they were HIV positive. The original survey was not designed to estimate HIV prevalence, so data on HIV serostatus were unreliable. Nonetheless, familiarity with HIV infection through awareness of one’s own or a friend/close relative’s serostatus was low (12%). Self-reported health-seeking behavior varied by type of healthcare sought: 76% reported lifetime use of government health facilities, 22% reported use of private pharmacies and 46% reported use of traditional healers. About one third believed that ART helps people with HIV to be healthier, and about 9% thought there were alternative treatments for HIV in the community, including from traditional healers.

**Table 1:** Characteristics of participants by VCT use indicators

**Variable**^**a**^	**Lifetime VCT Use**			**Past-6-Months VCT Use**			**Endorsement of VCT use**		
	**% never accessed**	**% accessed**	***P*-value**	**% never accessed**	**% accessed**	***P*-value**	**% not important**	**% important**	***P*-value**
	**(n = 3260)**	**(n = 489)**		**(n = 3375)**	**(n = 374)**		**(n = 393)**	**(n = 676)**	
Reside in isolated district^b^	41.0	37.5	<0.001	59.2	62.6	0.007	57.1	63.9	0.017
Understand Portuguese	36.6	49.6	<0.001	36.7	53.6	<0.001	42.6	49.0	0.002
Marital status			0.063			0.010			0.951
Married	74.9	69.8		73.7	75.9		73.5	71.3	
Divorced/separated	3.8	4.0		4.0	2.%		6.	3.3	
Single	17.0	19.1		18.0	13.9		16.7	18.1	
Widowed	4.3	7.1		4.4	7.5		3.5	7.3	
Religion			0.393			0.898			0.088
Catholic	45.0	41.4		45.4	37.6		42.7	44.7	
Protestant	13.7	20.1		13.8	21.6		10.5	16.8	
Evangelical/Pentecostal	15.7	20.2		16.1	19.1		14.1	16.4	
Other Christian^c^	3.9	6.2		3.8	7.5		5.2	8.3	
Muslim	9.9	4.4		9.7	4.0		14.2	4.8	
Non-Christian Eastern	2.9	1.0		2.7	1.2		1.7	1.9	
Other^c^	8.9	6.8		8.4	9.0		11.6	7.2	
HIV infection of self, relative and/or friend	8.0	25.2	<0.001	8.5	28.1	<0.001	9.8	24.9	<0.001
Accessed health facility	70.6	87.8	<0.001	71.7	87.0	<0.001	82.8	83.4	0.002
Accessed pharmacy	19.6	29.5	<0.001	19.8	31.6	<0.001	32.0	34.3	0.092
Accessed traditional healer	43.4	46.6	0.005	44.6	40.2	0.108	41.7	50.6	0.005
Believes ART helps people with HIV to be healthier	20.4	64.5	<0.001	22.7	65.1	<0.001	26.1	65.9	<0.001
Believes in alternative treatments for HIV	5.7	18.1	<0.001	6.3	19.0	<0.001	5.9	17.4	<0.001
Perceived chance of becoming infected with HIV			<0.001			<0.001			0.036
Don’t know	54.1	40.5		53.7	37.7		60.9	39.4	
No chance	25.1	15.8		24.1	18.6		15.3	22.2	
Small chance	15.3	26.4		15.4	29.7		14.7	23.1	
Good chance	4.8	9.6		5.8	5.3		5.5	9.9	
	**Median (IQR)**^**a**^	**Median (IQR)**		**Median (IQR)**	**Median (IQR)**		**Median (IQR)**	**Median (IQR)**	
Age (years)	29 (23–38)	26 (22–35)	<0.001	29 (23–38)	25 (22–33)	<0.001	28 (23–35)	26 (23–34)	0.004
Education (years)	1 (0–4)	3 (0–5)	<0.001	1 (0–4)	4 (0–5)	<0.001	2 (0–4)	3 (0–6)	<0.001
Distance to clinic (km)	7.2 (4.1 - 10.4)	4.3 (0.6-9.3)	<0.001	7.2 (3.9-10.4)	4.1 (0.6 - 9.3)	<0.001	6.1 (3–9.4)	4.6 (0.6-9.1)	0.006
Negative labeling and devaluation	38 (29–50)	33 (26–48)	0.496	38 (29–50)	37 (30–48)	0.859	44 (33–53)	38 (29–48)	0.788
Social exclusion	47 (33–67)	40 (27–56)	<0.001	47 (33–60)	40 (26–53)	<0.001	47 (32–67)	40 (27–53)	<0.001

[a] Continuous variables were analyzed as weighted estimates of medians (interquartile range), with each observation weighted by the inverse of the household sampling probability. Categorical variables were analyzed as weighted percentages, with each observation being weighted by the inverse of the household sampling probability. Tests of association with VCT outcomes (binary) were conducted using the rank sum test (continuous) or chi-square test (categorical).

[b] Isolated districts include Inhassunge, Milange, Morrumbala, Mopeia, Gile, Maganja da Costa and Lugela.

[c] ‘Other Christian’ includes LDS Mormon and Jehovah’s Witness. ‘Other’ includes Spiritual, Traditional Religions, and Agnostic or Atheist.

In unadjusted analyses (Table 1), participants who engaged in and/or positively endorsed VCT use generally had lower median stigma scores than participants who did not engage in and/or positively endorse VCT use. Differences in the medians for negative labeling/devaluation (NLD) scores were not statistically significant, but differences in the medians for social exclusion (SoE) scores were statistically significant.

### Correlates of VCT services uptake

Results for lifetime and past-6-months VCT use are presented in Table 2. In adjusted analyses, a 25-point increase (from 50 to 75) in the NLD score was associated with a 37% drop in odds for lifetime VCT use; that is, a 25-point decrease (from 75 to 50) in the NLD score was associated with 1.6-fold increase in lifetime VCT use (adjusted OR: 1.63, 95% CI: 1.14-2.32). A 50-point drop (from 100 to 50) in NLD was associated with a 2.7-fold increase in lifetime VCT use (adjusted OR: 2.68, 95% CI: 1.30-5.50). The relationship between the SoE score and lifetime VCT use was of marginal statistical significance (*p* = 0.097). Other exposures inversely associated with lifetime VCT use included age and distance to the nearest clinic. Positive associations were found for knowledge of HIV transmission routes, awareness of HIV infection, access/contact with public health facilities and private pharmacies, belief in ART efficacy, belief in the efficacy of alternative treatments for HIV and perceived risk of HIV infection. The perceived risk of HIV infection categories small chance, good chance and already infected each showed statistically significant odds of lifetime VCT use compared with the reference category (non-response/don’t know).

**Table 2:** Adjusted odds ratios and overall tests of significance for lifetime VCT use and past-6-months VCT use

	**Lifetime VCT use**			**Past-6-months VCT use**		
**Variable**	**OR**	**95% CI**	***P*-value**	**OR**	**95% CI**	***P*-value**
Negative labeling and devaluation (Ref = 50 pts)^a^			0.025			0.053
0 pts	1.05	0.62 – 1.78		1.25	0.70 – 2.24	
25 pts	0.87	0.72 – 1.05		0.94	0.76 – 1.15	
75 pts	1.63	1.14 – 2.32		1.63	1.10 – 2.41	
100 pts	2.68	1.30 – 5.50		2.69	1.21 – 5.98	
Social exclusion (Ref = 50 pts)^a^			0.097			0.006
0 pts	0.57	0.34 – 0.95		0.43	0.25 – 0.73	
25 pts	0.78	0.62 – 0.98		0.68	0.53 – 0.86	
75 pts	0.97	0.79 – 1.20		0.88	0.69 – 1.11	
100 pts	0.85	0.50 – 1.45		0.63	0.35 – 1.13	
Age (per 10 years)	0.83	0.73 – 0.94	0.002	0.84	0.74 – 0.96	0.01
Education (per 5 years)	1.21	0.94 – 1.55	0.14	1.15	0.88 – 1.50	0.3
Distance to clinic (per 5 km)	0.82	0.74 – 0.92	0.001	0.87	0.77 – 0.98	0.02
Reside in isolated district	1.02	0.79 – 1.33	0.9	1.02	0.76 – 1.36	0.9
Understand Portuguese	1.24	0.95 – 1.61	0.11	1.47	1.10 – 1.96	0.009
Marital status (Ref = married/common law)			0.3			0.065
Divorced/separated	0.63	0.32 – 1.24		0.28	0.11 – 0.74	
Single	1.18	0.90 – 1.55		1.07	0.80 – 1.45	
Widowed	1.14	0.67 – 1.94		0.87	0.47 – 1.64	
Religion (Ref = Catholic)			0.4			0.14
Evangelical and Pentecostal	1.16	0.84 – 1.61		1.02	0.70 – 1.49	
Muslim	0.90	0.59 –1.35		0.86	0.54 –1.37	
Non-Christian Eastern	1.61	0.83 – 3.10		1.69	0.85 – 3.34	
Other Christian	0.80	0.44 – 1.45		0.82	0.43 – 1.54	
Other	1.39	0.90 – 2.15		1.67	1.08 – 2.60	
Protestant	1.19	0.80 – 1.76		1.28	0.85 – 1.95	
Quality of life score (per 20 pts)	1.11	0.95 – 1.30	0.18	1.07	0.91 – 1.26	0.4
HIV knowledge score (8 versus 0 pts)	2.11	1.27 – 3.50	0.018	2.13	1.22 – 3.73	0.009
HIV infection of self, relative and/or friend	1.99	1.43 – 2.76	<0.001	1. 89	1. 34–2.67	<0.001
Accessed health facility	1.67	1.27 – 2.19	<0.001	1.38	1.03 – 1.85	0.03
Accessed pharmacy	1.39	1.07 – 1.82	0.014	1.46	1.09 – 1.94	0.01
Accessed traditional healer	1.05	0.84 – 1.32	0.7	0.96	0.75 – 1.23	0.7
Believes that ART helps people with HIV to be healthier	3.46	2.65 – 4.52	<0.001	2.98	2.21 – 4.00	<0.001
Believes in alternative treatments for HIV	2.08	1.48 – 2.92	<0.001	2.18	1.53 – 3.11	0.0002
Perceived chance of becoming infected with HIV (Ref = non-response/don’t know)			<0.001			<0.001
Already infected	5.15	2.42 – 11.0		4.00	1.90 – 8.43	
Good chance	2.27	1.54 – 3.35		1.77	1.14 – 2.76	
Small chance	1.71	1.28 – 2.29		1.57	1.14 – 2.15	
No chance	0.97	0.71 – 1.31		1.12	0.81 – 1.54	

*NLD* Negative labeling and devaluation; *SoE* Social exclusion; *OR* odds ratio, *CI* confidence interval. If the 95% CI excluded 1, then there was a significant pairwise association between that category and the outcome. There was little evidence of an interaction between NLD and SoE with lifetime VCT use or past-6-months use (*p* > 0.6).

[a] NLD, SoE and HIV knowledge scores were modeled using restricted cubic splines with 4 knots.

Both the NLD and SoE stigma scores were associated with low odds of past-6-months VCT use. An NLD score of 75 versus 50 resulted in 63% higher odds of past-6-months VCT use (adjusted OR: 1.63, 95% CI: 1.10-2.41). A SoE score of 25 versus 50 (i.e., 25-point increase) was associated with a 32% decrease in past-6-months VCT use (adjusted OR: 0.68, 95% CI: 0.53-0.86). That is, a 25-point decrease in the SoE score from 50 to 25 was associated with 1.5-fold increase in odds of past-6-months VCT use. A 50-point decrease in the SoE score from 50 to 0 was associated with 2.3-fold increase in odds of past-6-months VCT use. All the other predictors of lifetime VCT use also predicted past-6-months VCT use, except for fluency in Portuguese, which was positively and significantly associated with past-6-months VCT use (*p* = 0.009), but was not associated with lifetime VCT use (*p* = 0.11).

### Correlates of the view that “It is worthwhile to receive VCT and learn your HIV status”

Table 3 presents results for perceived importance of VCT and learning about one’s HIV status. A high NLD stigma score was associated with low odds of endorsing the statement, “It is worthwhile to receive VCT and learn your HIV status” (adjusted OR: 0.94, 95% CI: 0.77-1.15, *p* = 0.04). The SoE stigma score showed an even stronger inverse association (adjusted OR: 0.75, *p* < 0.001). That is, a 25-point decrease in the SoE score was associated a 1.3-fold increase in odds of endorsing VCT. Similarly, scores for QoL-chronic, HIV knowledge, quality of last VCT service accessed, contact with traditional healers and belief in ART efficacy were each associated with higher odds of endorsing VCT. Living in an isolated district compared with not living in an isolated district was associated with lower odds of endorsing VCT (*p* = 0.047). When comparing participants who knew about the existence of VCT services with those who did not, awareness of VCT was related to lower endorsement of SoE, but not to endorsement of NLD.

**Table 3:** Adjusted odds ratios and overall tests of significance for endorsing VCT use

	**It is worthwhile to receive VCT and learn your HIV status**		
**Variable**^**a**^	**OR**	**95% CI**	***P*-value**
Negative labeling and devaluation (Ref = 50 pts)^a^			0.04
0 pts	1.14	0.68 – 1.91	
25 pts	1.07	0.83 – 1.38	
75 pts	0.94	0.72 – 1.21	
100 pts	0.88	0.52 – 1.47	
Social exclusion (Ref = 50 pts)^a^			<0.001
0 pts	1.96	1.23 – 3.10	
25 pts	1.40	1.11 – 1.76	
75 pts	0.71	0.56 – 0.90	
100 pts	0.51	0.32 – 0.81	
Age (per 10 years)	0.91	0.76 – 1.08	0.3
Education (per 5 years)	0.91	0.62 – 1.34	0.6
Distance to clinic (per 5 km)	0.96	0.81 – 1.14	0.6
Reside in isolated district	0.68	0.47 – 0.99	0.047
Understand Portuguese	0.95	0.65 – 1.40	0.8
Marital status (Ref = married/common law)			0.8
Divorced/separated	1.00	0.41 – 2.43	
Single	0.83	0.55 – 1.25	
Widowed	1.00	0.48 – 2.07	
Religion (Ref = Catholic)			0.5
Evangelical and Pentecostal	0.73	0.44 – 1.19	
Muslim	1.15	0.67 – 1.97	
Non-Christian Eastern	0.64	0.23 – 1.76	
Other Christian	0.54	0.24 – 1.22	
Other	0.84	0.47 – 1.48	
Protestant	1.15	0.64 – 2.06	
Quality of life score (75 versus 50 pts)	3.04	1.95 – 4.76	<0.001
HIV knowledge score (5 versus 1 pt)	1.68	1.06 – 2.68	0.047
HIV infection of self, relative and/or friend	1.02	0.62 –1.69	0.9
VCT experience (Ref = Never tested)			<0.001
Ever tested & no test results received	2.79	1.64 – 4.73	
Ever tested & results received	7.61	5.08–11.4	
Accessed health facility	1.27	0.86 – 1.87	0.2
Accessed pharmacy	0.87	0.59 – 1.29	0.5
Accessed traditional healer	1.63	1.17 – 2.28	0.004
Believes that ART helps people with HIV to be healthier	6.47	4.36 – 9.59	<0.001
Believes in alternative treatments for HIV	1.04	0.59 – 1.83	0.2
Perceived chance of becoming infected with HIV (Ref = non-response/don’t know)			0.9
Already infected	1.41	0.41 – 4.79	
Good chance	1.20	0.66 – 2.17	
Small chance	1.07	0.68 – 1.68	
No chance	1.64	1.08 – 2.49	

95% CI excluded 1, then there was a significant pairwise association between that category and the outcome.

[a] An interaction term between NLD and SoE was included (p = 0.014); estimates were adjusted to median values (NLD = 41 and SoE = 47). NLD and SoE are modeled as linear (no evidence of nonlinearity).

## Discussion

In this study of the relationships between stigma (negative labeling and devaluation (NLD) and social exclusion (SoE) stigmas) and the use and endorsement of voluntary counseling and testing (VCT) services, the importance of VCT was acknowledged by more than half of the study participants (63%), but actual use was low (only 13% reported lifetime VCT use). Similarly low levels of VCT use have been reported in other studies from Sub-Saharan Africa [32,33]. Thus, there is a need to investigate barriers to VCT use among individuals who have favorable opinions about the importance of testing for HIV infection in Zambézia Province and other parts of Sub-Saharan Africa.

In general, stigma seems to have a negative association with VCT use and the perceived importance of VCT use. This association is independent of associations with knowledge about HIV transmission and treatment, perceived risk of HIV infection, familiarity with HIV infection, healthcare services access/contact, health-related quality of life, belief in ART efficacy and structural barriers like distance to a clinic and delays in disseminating HIV test results to VCT users. Thus, unraveling the specific aspects of stigma that impede VCT use and the complex intersections among them is needed. These data suggest that the relationship between endorsement of community stigma and VCT uptake depends on the domains of the stigma under consideration. In particular, endorsement of SoE was associated with low odds of past-6-months VCT use and low perceived importance of VCT, but was not associated with lifetime VCT use. Endorsement of NLD of people living with HIV/AIDS (PLWHA) was associated with significantly lower odds of lifetime VCT use and marginally lower odds of past-6-months VCT use, but was not associated with perceived importance of VCT. Since awareness of VCT was related to lower SoE, but not NLD, it is plausible that acquiring information about VCT (or exposure to such information) mitigates the tendency to socially exclude PLWHA, whereas negative stereotypes of PLWHA might be more entrenched in the community and less amenable to change with increased knowledge of VCT. Due to nonlinearity, the impact of changes in stigma scores that we report upon (i.e. 25 and 50 point changes) might not accurately reflect the impact of smaller changes on the stigma scales. By having Figure 1, the reader should be able to judge the relationship for the whole range of values that the stigma scales may take on.

Nonetheless, evidence regarding the domain-specificity of stigma and its effect on VCT use and endorsement is somewhat weak. In fact, the potential effects of NLD stigma on VCT use and endorsement were only evident in adjusted analysis, and the effects of SoE, though relatively robust, were confounded by other factors. These two domains of community stigma seem to have smaller effect sizes than the effects of the other covariates considered. Therefore, it is important to consider the effects of some of the covariates examined in this study.

Most of our findings about correlates of VCT use and endorsement are consistent with the literature on VCT from various settings [2,32,33]. For example, VCT use is less likely among older people than among younger people, with an increase in distance between the nearest health facility and one’s place of residence and/or with a decrease in perceived risk of HIV infection. Our data suggest that the quality of past testing is also important. In particular, receiving versus not receiving HIV test results was associated with perceived importance of VCT services. Compared with participants who had never been tested, the adjusted OR for endorsing VCT use was 2.71 among those who had tested but had not received their HIV test results in the past and was 7.33 among those who had both tested and received their results. Thus, organizational factors such as efficient and reliable communication of test results should not be overlooked in studies investigating patient- and community-level barriers to VCT use such as community stigma. In addition, greater knowledge of HIV transmission routes predicted lifetime VCT use, past-6-months VCT use and positive attitudes towards VCT. Increased distance between one’s residence and the nearest public health facility was associated with less VCT use (lifetime and past-6-months), but not with attitudes towards VCT. This is important because, based on age and gender, our participants are likely to have higher than average contact with the public health system or at least have high incentives to seek public healthcare, yet distance, in addition to stigma, might impede their efforts to seek healthcare services such as VCT.

A key limitation of this study is its cross-sectional design, which precludes examination of causality or temporal effects. While we were able to distinguish two distinct and internally consistent stigma constructs, our measure might have missed other important domains of stigma. The stigma measure could be further refined and validated in subsequent studies by using confirmatory factor analysis techniques. In addition, small effect sizes for stigma could be due to measurement error. The need for better and more reliable measures of stigma is widely acknowledged in the field [8,13,34]. Future research should explore these issues.

## Conclusion

Stigma reduction in general, in addition to efforts to address other known barriers, could have a positive impact on VCT services uptake. VCT use and endorsement might differ by domains of community stigma. However, this needs to be further investigated, as does the potential for domain-specific stigma reduction interventions. In tandem with stigma reduction efforts, making VCT services more accessible by moving them closer to communities and improving the quality of VCT services, such as by ensuring that testers get their results, could also promote greater uptake and favorable disposition towards VCT services among female heads of household in Zambézia Province, Mozambique.

## Abbreviations

HIV/AIDS: Human immunodeficiency virus/acquired immune deficiency syndrome; NLD: Negative labeling and devaluation stigma; PLWHA: People living with HIV/AIDS; SoE: Social exclusion stigma; VCT: Voluntary counseling and testing.

## Competing interests

The authors declare that they have no competing interests.

## Authors’ contributions

AM made substantial contributions to the study conception and design, data analysis plan, interpretation of data and drafted the manuscript. MB made substantial contributions to design, acquisition of data, analysis and interpretation of data, drafting the methods sections of the manuscript and revising the whole manuscript critically for important intellectual content. BV made substantial contributions to the study design, acquisition and interpretation of data, and revised the manuscript critically for important intellectual content. HNP, LV and MS made substantial contributions to the interpretation of data and revised the manuscript critically for important intellectual content. AV made substantial contributions to design, acquisition of data, interpretation of data and revising the manuscript critically for important intellectual content. All authors approved the version to be published.

## Pre-publication history

The pre-publication history for this paper can be accessed here:

http://www.biomedcentral.com/1471-2458/13/1155/prepub

## Supplementary Material

Additional file 1 List of stigma, HIV/AIDS knowledge and quality of life questions. Click here for file
