# Supplementary material for: Community stigma endorsement and voluntary counseling and testing behavior and attitudes among female heads of household in Zambézia Province, Mozambique
Source: BMC Public Health. 2013 Dec 10;13:1155. doi: 10.1186/1471-2458-13-1155 (PMC4029405; doi:10.1186/1471-2458-13-1155)
Supplement: Additional file 1 — List of stigma, HIV/AIDS knowledge and quality of life questions. [file 1471-2458-13-1155-S1.docx]

**Additional file 1**

**Quality of Life**

| 1. How would you rate your quality of life? 2. How satisfied are you with your health? 3. To what extent do you feel that physical pain prevents you from doing what you need to do? |
| --- |
| 1. How much do you need medical treatment to function in your daily life? |
| 1. Do you have enough energy or vitality for everyday life? |
| 1. How satisfied are you with your ability to perform your daily living activities? |
| 1. How satisfied are you with your capacity to work? |
| 1. How satisfied are you with your access to health treatment? |
| 1. How easy is it to get where you want to go? |
| 1. How often do you have negative feelings such as despair, anxiety, or depression? |

**HIV Knowledge**

| 1. In what ways can one adult man or woman transmit HIV to another man or woman? |
| --- |
| 1. In what ways can a woman with HIV pass it to her baby? |
| 1. How can HIV transmission from an adult man or woman to another be prevented? |
| 1. How can HIV transmission from mother to a child be prevented? |
| 1. Do you think there is a cure for HIV/AIDS? |

**Community Stigma Questions**

| ***Negative Labeling & Devaluation*** (α = 0.742) |
| --- |
| A person who has AIDS should not be allowed to work with other people to protect the people who don’t have AIDS. |
| A person who has AIDS should not be allowed to make food to sell (to be consumed by other people). |
| AIDS is a punishment for bad behavior. |
| AIDS is a punishment from God. |
| People with HIV/AIDS should be marked so everyone could identify them. |
| Almost all people who have HIV/AIDS are prostitutes or sexually immoral. |
| If you learned that a friend of yours had AIDS, you would stop being their friend. |
| If you told your regular partner that you have HIV/AIDS, s/he would leave you. |
| If you had AIDS, people would avoid you |
| ***Social Exclusion*** (α = 0.731) |
| It is better not to hide that you have AIDS, so you can get support from friends or family. |
| You would feel comfortable living closely with someone who has HIV/AIDS |
| You would be willing to care for a relative with AIDS in your house/home. |
| If you saw someone with HIV/AIDS being mistreated, you would try to help him or her. |
| It is safe to let your child play with children who have HIV/AIDS. |
| You would worry about touching someone with HIV/AIDS. |

[a] Answers were coded 1-4 with highest stigma as 4. Each scale is the mean score of all non-missing items, normalized to range 0-100.

α = Cronbach’s alpha, a measure of the reliability of the factor/scale based on the internal consistency of the constituent items.
